# Validation of a QTL associated with resistance to *Vibrio anguillarum* in rainbow trout (*Oncorhynchus mykiss*)

**DOI:** 10.1186/s13028-023-00692-z

**Published:** 2023-06-26

**Authors:** Asma Mohammad Karami, Moonika Haahr Marana, Heidi Mathiessen, Inger Dalsgaard, Torben Fejer Nielsen, Per Walter Kania, Kurt Buchmann

**Affiliations:** 1 Department of Veterinary and Animal Sciences, Faculty of Health and Medical Sciences, University of Copenhagen, DK-1870 Frederiksberg C, Denmark; 2 National Institute of Aquatic Resources, Technical University of Denmark, DK-2800 Kgs. Lyngby, Denmark; 3 Aquasearch ova ApS, Hedegaardsvej 8, DK-7190 Billund, Denmark

**Keywords:** Fish, Marker associated selective breeding, Single nucleotide polymorphism, SNP, Vibriosis

## Abstract

Vibriosis is a bacterial disease in fish caused by the Gram negative bacterium *Vibrio anguillarum* with severe impact on rainbow trout (*Oncorhynchus mykiss*) farming. Sustainable control methods should be developed and we here show that marker assisted selective breeding of fish naturally resistant to the disease is feasible. We have validated the use of a single nucleotide polymorphism (SNP) marker SNP AX-89,945,921 (QTL on chromosome 21). The QTL was previously found associated with resistance to vibriosis and described following a genome wide association analysis (GWAS) of trout exposed to the bacterium. For this validation spawners were genotyped by use of the 57 K Axiom®Trout Microarray (Affymetrix) and homozygous male fish carrying the allele with the SNP AX-89,945,921 were then selected and used to fertilize eggs from outbred female trout resulting in fish all carrying the SNP (QTL-fish). Control fish (non-QTL fish) were produced by fertilizing the same batch of eggs by use of male parents negative for the SNP. The fish were exposed in freshwater to *V. anguillarum* (water bath infection) at 19 C°. A total of 900 fish were challenged in a common garden set-up in triplicate. A bacterial solution of *V. anguillarum* (serotype O1) was added to each of three freshwater fish tanks, each with 150 QTL and 150 non-QTL fish. Fish were tagged by tail fin cut (upper/lower) to discern the two groups, whereafter fish were monitored around the clock to detect disease signs and remove moribund fish. Clinical vibriosis developed within two days in non-QTL-fish (overall morbidity of 70%). QTL fish developed clinical signs later and the morbidity was significantly lower and did not reach 50%. Rainbow trout farming may benefit from using the QTL associated with higher resistance towards vibriosis. The effect may be optimized in the future by use of both male and female parents homozygous for the marker allele.

## Findings

*Vibrio anguillarum* is a Gram negative bacterium causing vibriosis in a range of wild and cultured fish species including salmonids [1]. Maricultured rainbow trout in Danish waters suffer from the disease throughout the production period [2], and although a commercial trivalent vaccine is available it is not fully protective under field conditions [3]. An alternative approach to reach control of disease in aquacultured fish is based on selective breeding of naturally resistant fish [4]. The application of genome wide association analysis (GWAS) and search for genetic markers has lifted the breeding efficacy significantly [5]. This has proven successful for identification of genomic markers in fish associated with higher resistance towards viral diseases [6–10], various bacterial diseases [11–13] and parasitic diseases [14, 15]. A genetic background for vibriosis resistance has been reported for Japanese flounder (*Paralichthys olivaceus*) [16, 17], tongue sole (*Cynoglossus semilaevis*) [18] and turbot (*Scophthalmus maximus*) [19], and we recently showed that *V. anguillarum* resistance in rainbow trout (*Oncorhynchus mykiss*) is associated with SNPs on rainbow trout chromosome 21 (Omy21) [20]. In order to validate this finding and seeking a practical application of the knowledge we have performed a comparative challenge study exposing trout with high and low frequencies of the SNP. We produced QTL-fish by fertilizing rainbow trout eggs with sperm from three brood stock males, which were homozygous for the SNP AX-89,945,921 (associated with *V. anguillarum* resistance [20]). Non-QTL-fish were produced by fertilizing eggs from the same batch with sperm from three males negative for the SNP. The eggs were from 30 outbred female spawners carrying the SNP AX-89,945,921 at a low frequency, 7% homozygous and 44% heterozygous for the SNP associated with resistance. It was estimated that the frequency of heterozygotes in the non-QTL fish (fertilized by negative males) was 29% and that 71% of the non-QTL fish were fully negative for the SNP. QTL fish group comprises 29% homozygotes and 71% heterozygotes. The typing was achieved by use of the 57 k SNP chip (Axiom®Trout, Affymetrix, San Diego, United States) [21].

The eggs from the breeding station were hatched and reared at the disease-free recirculated hatchery (Bornholm Salmon Hatchery). The fish population delivering eggs for the validation trial differed from the population delivering eggs for the original QTL study [20]. Thus, no genetic exchange had occurred for decades between these farms, Fårup Mølle in Jelling and Refsgaard in Egtved (Jutland, Denmark), respectively. The fish were fed by pelleted feed (Inicio, Biomar, Denmark). When reaching a mean body weight of 6 g fish (1200 degree days) the fish were transported (3 h) in oxygenated cooled freshwater to the infection facility at the University of Copenhagen and acclimated at 19 °C. Full survival was confirmed after transport. Before experimentation fish were acclimatized and tagged in the infection facility. For acclimatization and tagging fish were placed in separate fish tanks with aerated and recirculated (internal filters, Eheim, Germany) freshwater at 19 °C for one week. In order to differentiate fish during their pathogen exposure in a common garden set-up, we tagged the fish during the acclimatization week by tail cut under anaesthesia (immersion into 50 mg/L Tricaine Methane Sulphonate MS222, Sigma-Aldrich, Denmark). Fish carrying a high frequency of the SNP associated with resistance (QTL fish) had their upper part of the tail fin cut. Non-QTL fish, with a low frequency of the SNP, had their lower tail fin cut.

Fish (mean weight 6 g, mean length 8 cm) were exposed at 19 °C to *V. anguillarum.* We used the same bacterial isolate (serotype O1, isolate 130,829–1/3) as applied for the challenge of rainbow trout on which the original GWAS and QTL description was based [20]. The bacterium was cultured in KB-medium over 72 h at 20 °C, and the fish were exposed by adding a solution (2.4 × 10^9^ colony forming units (cfu)/mL) of the bacterium to the fish tanks. A common garden set-up was applied, whereby both QTL-fish and non-QTL fish were kept in the same fish tank securing that the two types of fish were exposed to similar environmental conditions. Fish were exposed for 2 h in triplicate aerated 100 L fish tanks (with a reduced water volume of 30 L) with 2 × 150 fish in each with a final *V. anguillarum* concentration of 3.12 × 10^7^ cfu/mL. Following exposure water was replenished to 100 L and two internal Eheim biofilters were placed in each tank. The fish were kept in constant light during the entire period of observation. Control fish in three fish tanks were kept in a separate but similar room under corresponding conditions but without being exposed to *V. anguillarum.*

The rainbow trout were monitored around the clock by checking the fish every second hour. In case moribund fish, showing severe clinical signs (equilibrium disturbance, exophthalmia, skin ulcers), were noted, the affected fish were removed from the trial and euthanized in an over dosage of MS222 (Tricaine Methane Sulphonate 300 mg/L) and recorded as mortality. Head kidney swabs of the diseased fish were inoculated on blood-agar plates for confirmation of the causative pathogen [2].

Mortality curves for the six different groups were prepared by the Graph Pad Prism version 9.50. Data were first tested for normality by the D’Agostino and Pearson test. A two tailed paired Student’s t-test using a probability level of 1% (0.01) was applied to test for differences between groups. First step was to compare mortality rates between replicates within both the non-QTL group and within the the QTL-group. Finally the non-QTL fish groups were tested against the QTL fish groups (both for the replicates and the combined groups).

**Fig. 1:**
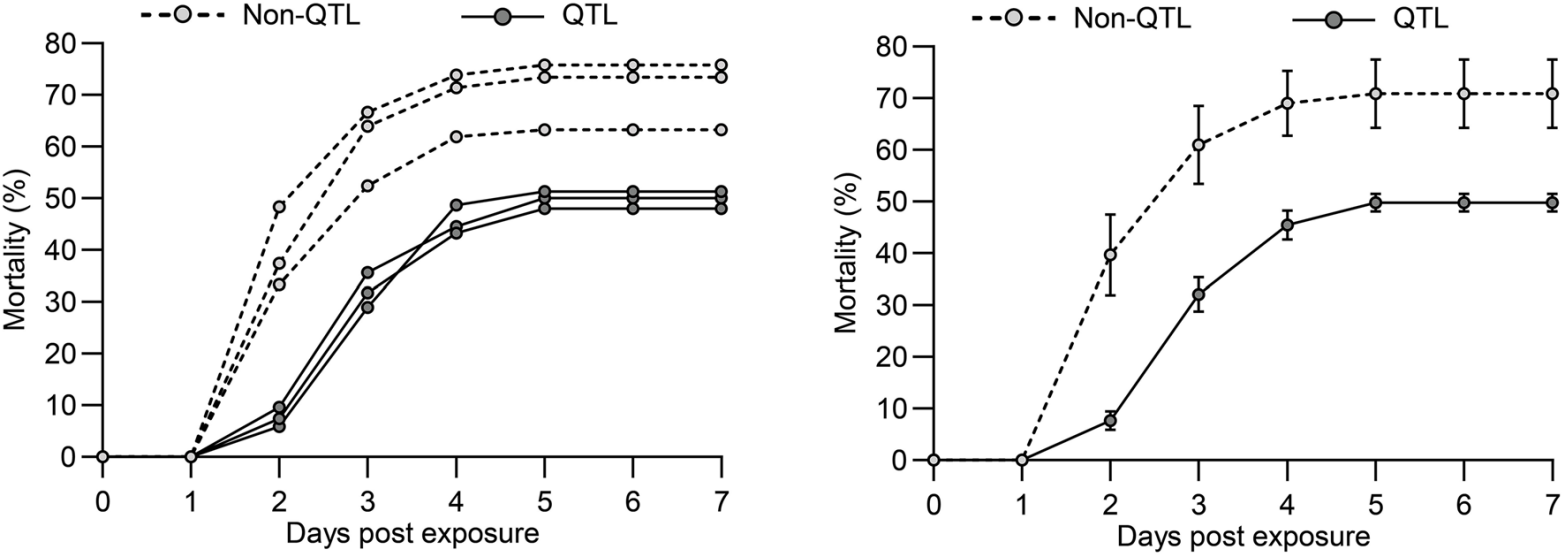
Left: Mortality curves (triplicate trials) for QTL fish and non-QTL fish following bath exposure to *V. anguillarum*. Right: Mortality curves (trials combined) for QTL fish and non-QTL fish following bath exposure to *V. anguillarum.* Mean percentage and SD shown

The first disease signs were observed on day 1 post-exposure (1 dpe) in one specimen of the non-QTL fish and from 2 dpe the number of diseased fish in this group increased markedly in all triplicates. Morbidity in non-QTL fish accelerated at 2 dpe before noon, where only one QTL-fish but 34 non-QTL fish died. Morbidity decreased later at 4 dpe and the curve flattened on 5 dpe, whereafter the experiment was terminated on 7 dpe. Thus, the percentage of fish affected in the three non-QTL-groups reached 61, 70 and 72% (Fig. 1, left). This mortality level was for all non-QTL groups significantly higher compared to the QTL groups (P < 0.01). The QTL-fish showed a significantly lower disease rate and in none of the groups the overall percentage of fish with clinical signs surpassed 50% (Fig. 1, left). Within the two groups (QTL and non-QTL fish) the subgroups did not differ significantly, and when combined within groups the non-QTL fish showed an over-all morbidity rate of 70%, whereas the QTL-fish remained at 49% (Fig. 1, right). Also, when combined the morbidity rates were significantly lower in QTL-fish compared to non-QTL fish (P = 0.0035). Control fish which were kept without *V. anguillarum* exposure showed no disease signs and mortality at all.

Vibriosis is considered an important disease problem in rainbow trout farming but even with vaccines aquaculture enterprises may benefit from use of naturally resistant fish strains [4, 20]. Breeding of disease resistant fish leads to improved health of aquacultured fish, which again will increase survival, improve feed efficiency and reduce usage of medicines such as antibiotics. Convincing results have been presented for Atlantic salmon (*Salmo salar*) with regard to viral diseases, including infectious pancreatic necrosis virus (IPNV) and salmonid alpha virus (SAV) [6, 7, 9, 10]. Loci associated with disease resistance in rainbow trout have been presented against the bacterium *Flavobacterium psychrophilum* [11–13], the bacterium *V. anguillarum* [20] and the parasite *Ichthyophthirius multifiliis* [14, 15]. Other studies suggested loci for vibriosis resistance in flatfish, such as Japanese flounder [16, 17], tongue sole [18] and turbot [19]. This study has validated the QTL for vibriosis resistance in trout. We performed controlled infection experiments and confirmed that the QTL for natural resistance against *V. anguillarum* described by Karami et al. [20] may find a practical application and be used for selective breeding of resistant fish strains. It is noteworthy that the rainbow trout populations used for description of the QTL [20] is genetically different from the population used for validation in this study. No exchange of genetic material between these farms (Fårup Mølle and Refsgaard trout farms) has taken place for decades, which suggests a generic application of the QTL. We showed in a triplicate and controlled study that morbidity and thereby expected mortality could be reduced from 70% to less than 50% simply using fish with male parents homozygous for the SNP AX-89,945,921. The QTL offspring produced comprised both homozygous (estimated to 29%) and heterozygous trout (estimated to 71%). It should therefore be investigated if production of a batch of fully homozygous offspring can increase the natural resistance to *V. anguillarum*. In the present study we merely used homozygous male parents and outbred female spawners. Selection of both female and male spawners homozygous for the SNP is likely to increase the resistance of the rainbow trout offspring. We therefore hypothesize that the natural resistance of those fish towards vibriosis would be even higher. It is recommended to evaluate this approach in a future study. In this context genetic variance and dominance effects should considered as well. Future experiments should therefore produce fully homozygous offspring by using male and female parents homozygous for the allele and evaluate their susceptibility/resistance to the vibriosis.

## Data Availability

The datasets used and/or analysed during the current study are available from the corresponding author on reasonable request.

## Abbreviations

GWAS: Genome wide association analysis
IPNV: Infectious pancreatic necrosis virus
Omy21: *Oncorhyncus mykiss* chromosome 21
QTL: Quantitative trait loci
SAV: Salmonid alpha virus
SNP: Single nucleotide polymorphism
